# Reproducibility of pulmonary function tests in patients with systemic sclerosis

**DOI:** 10.1038/s41598-023-45881-y

**Published:** 2023-11-03

**Authors:** P. Jacquerie, B. André, D. De Seny, M. Henket, L. Giltay, M. Ernst, R. Louis, M. Malaise, C. Ribbens, J. Guiot

**Affiliations:** 1 Rheumatology Department, CHU LiègeULiège, Domaine Universitaire Sart Tilman, B35, 4020 Liège, Belgium; 2 Pneumology Department, CHU Liège, GIGA Research, ULiège, Domaine Universitaire Sart Tilman, Liège, Belgium; 3 Biostatistics and Research Method Center (B-STAT), ULiège, Domaine Universitaire Sart Tilman, Liège, Belgium

**Keywords:** Systemic sclerosis, Respiration

## Abstract

Systemic sclerosis (SSc) is a rare autoimmune disease in which interstitial lung disease (ILD) is the leading cause of morbidity and mortality. Clinical management of the lung disease is mainly based on pulmonary function testing (PFT) and their changes over time. Little is known about the reproducibility of PFT testing in SSc patients. The aim of this study was to assess the test–retest reliability and reproducibility of PFTs in SSc patients with or without ILD over 30 days in order determine the potential physiologic variation over the time. We performed prospective observational study of SSc patients. The FVC, FEV1/FVC ratio, DLCO and KCO parameters were assessed in this population at four different timepoints; T0 (time 0) and H3 (T0 + 3 h) defined test–retest reliability, D15 (T0 + 15 days) and D30 (T0 + 30 days) for reproducibility. A mixed linear model was used to test the effect of time (and therefore reproducibility) on patients and we looked for an interaction. We included 25 SSc patients divided in two groups, 14 with ILD and 11 non-ILD. Interactions between time and group were not significant and were not reported. Time and group did not significantly influence the different measures of the PFT: FVC [p values time and group effect respectively (0.33; 0.34)], FEV1/FVC ratio (0.093; 0.056) and DLCO (0.99; 0.13) in the ILD and non ILD group (Table S2). The analyse with interactions between time and group were not significant and are not reported. We also used a Bland Altman test to assess reproducibility for FVC (L) and DLCO (mMKpa/min/L), Figs. 1 and 2 respectively. The measurements were therefore reproducible over time and in each group. PFT parameters are reproducible over time in a clinically stable population of SSc (no significant effect of the time T0, H3, D15 and D30) and there is no significant distinction between patients with ILD and no ILD. These respiratory functional data can further underline their use in clinical practice.

## Introduction

Systemic sclerosis (SSc) is a potentially serious and disabling connective tissue disease affecting multiple organ systems, including the lungs^1^. Interstitial lung disease (ILD) is the leading cause of death in SSc.

Its accepted physiopathological pattern associates dysimmunity^2,3^, vasculopathy^4^ and fibrotic process on a background of genetics and environmental factors. Epigenetic modifications in fact modulate its pathogenesis, which associates profound immuno-inflammatory deregulation, abnormal behavior of endothelial cells and cellular trans-differentiation into myofibroblasts responsible for excessive deposition of extracellular matrix. Other cell types of probable pathogenic importance are pericytes, platelets and keratinocytes in conjunction with their relationship to vascular wall cells and fibroblasts^5^.

Pulmonary function test (PFTs) are non-invasive tools for investigating pulmonary function and are effective in the early detection of SSc-related lung damage, the assessment of their severity and the prediction of mortality^6^. Their usability has been previously validated in SSc monitoring, ideally using a combination of parameters. Indeed, PFT can explore lung function through the volume evaluation with forced vital capacity (FVC), total lung capacity (TLC) and alveolo-capillary function with diffusing lung capacity of CO (DLCO). The most common parameters used in the longitudinal follow up are mainly FVC and DLCO^7–9^. Indeed, whereas FVC can be reduced due to the progression of SSc associated interstitial lung disease (SSc-ILD), the reduction of DLCO can precede the acute inflammatory process or reflect the progression of ILD as well as the occurrence of PAH^10^. PFTs associated with high resolution thoracic computed tomography (HRCT) damage help to define a prognosis for SSc-ILD (limited or extensive disease)^11^.

In the current practice, PFTs are classically performed regularly (every 3–6 months or even more frequently in case of rapidly progressive lung disease or in order to quantify treatment response) allowing functional longitudinal monitoring^10^.

However, the inter- and intra- test variability of measurements in general population and in patients with preexisting respiratory conditions (e.g., asthma, COPD and cystic fibrosis) is well known and reduced as much as possible via strict protocols^12–14^.

To the best of our knowledge we prospectively explore for the first time the potential intra-individual variation of PFT in patients suffering from SSc-ILD. We confirm the good reliability of PFT and particularly for FVC and DLCO in this specific population both for patients suffering from SSc with or without ILD. This study was perform on a short and mid-term re-assessment (T0, T0 + 3H, D15 and D30) confirming its stability over the time in a clinically stable population.

## Methods

We prospectively analyzed PFT of patients at different timepoints. The diagnosis of SSc was made according to the international recommendations of the ACR/EULAR for the classification of systemic sclerosis^15^ and the distinction of the cutaneous forms into limited and diffuse according to the classification of Leroy et al.^16^. Early forms of SSc, also called limited, have been classified according to Leroy and Medsger^17^ while sine scleroderma fulfilled Poormoghim criteria^18^. The protocol was approved by ethics committee of CHU Liège, all subjects gave written informed consent before their enrolment (Belgian number: B7072020000033) and we confirm that all experiments were performed in accordance with relevant guidelines and regulations.

Patients were recruited on a voluntary basis during the period of June 2020 to September 2020 by consecutive method. They were seen at each visit by a physician who assessed the absence of acute intercurrent pathology that could disrupt the measure of the PTFs. Patients with infection, intercurrent disease, a change in their treatment, disease acute progression or lack of compliance with PFTs were excluded from the study. In addition, the selected patients had stable respiratory function for 1 year (monitorised every 3 months). The same examiner and spirometric system were used for test–retest reliability (T0, H3) and reproducibility (D15, D30). The Shapiro–Wilk test was used to assess the normal distribution of the parameters.

### Pulmonary function tests

Lung function tests were performed in our routine respiratory laboratory at CHU Liège in accordance with the recommendations of the European Respiratory Society (ERS)^19^. Results were expressed in ml and as percentage of predicted normal values. The Tiffeneau index or FEV-1/FVC is expressed as percentage. The diffusion capacity of CO (DLCO) and carbon monoxide transfer coefficient (KCO or DLCO/VA) were measured by the single breath testing technique (Sensor Medics 2400 He/CO Analyzer System, Bilthoven, The Netherlands).

### Statistical analysis

**Figure 1:**
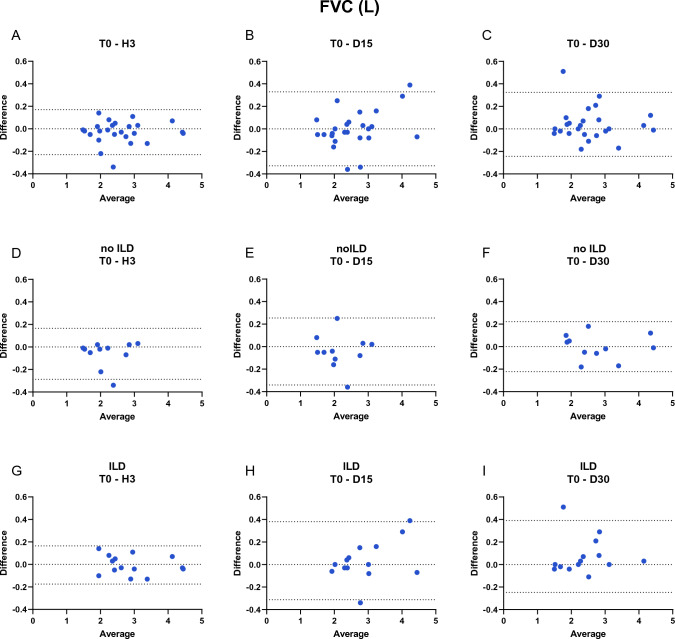
BLAND ALTMAN test FVC (L). Graphs (**A**–**C**) are created using data from all patients, graphs (**D**–**F**) are created using data from non-ILD SSc patients and graphs (**G**–**I**) are created using data from -ILD SSc patients. Plots (**A**,**D**,**G**) represent the difference between FVC (L) at T0 and H3 vs mean of FVC (L) at T0 and H3. Plots (**B**,**E**,**H**) represent the difference between FVC (L) at T0 and D15 vs mean of FVC (L) at T0 and D15. Plots (**C**,**F**,**I**) represent the difference between FVC (L) at T0 and D30 vs mean of FVC (L) at T0 and D30. Dotted line represent the difference 0 and the 95% of limit agreement of difference between the two measurements.

The variables are described at each time point, using means, standard deviations (SD), quartiles and extremes. The graphs represent the evolution of measurements over time (means and standard errors, SE). A mixed linear model is used to test the effect of time (and therefore reproducibility) and group. The presence of an interaction was also tested. A p < 0.05 was considered as significant. We presented the results via Bland Altman method for more visibility (Fig. 1).

Calculations using SAS version 9.4 and graphics using R version 3.6.1.

## Results

The demographic, functional and treatment characteristics of the subjects are given in Table 1. Respiratory function in the ILD/no-ILD subgroups is described in Table S2 (supplementary materials).

**Table 1:** Subjects characteristics.

	SSc no-ILD	SSc ILD
	(n = 11)	(n = 14)
Age, years	60 ± 14	66 ± 14
Gender (M/F)	1/10	2/12
BMI, kg/m^2^	25.9 ± 5.2	25.1 ± 3.5
Smokers (NS/FS/S)	9/2/0	12/0/2
Immunosuppressor yes/no	4/7	8/6
OCS yes/no	3/8	7/7
Disease duration in years	8.2 (12–3)	4.71 (4–3)
Rodnan skin score	3 (6–0)	3.5 (6–0)
ACR/Eular score	10 (17.5–8)	12.5 (19–7)
Limited SSc/lcSSc/dcSSc/sine scleroderma (n)	3/8/0/0	0/7/2/5
Musculoskeletal involvement	0	0
Renal crisis	0	0
Cardiac involvement	0	0
Pulmonary arterial hypertension (PAH) (n)	1	2

Data are expressed in mean ± standard deviation when the distribution is normal and in median (interquartile range) when the distribution is non-parametric. No significant difference was observed between the two groups (p > 0.05).

*lcSSc* limited cutaneous systemic sclerosis, *dcSSc* diffuse cutaneous systemic sclerosis.

A total of 25 participants SSc were finally included in this study: 14 with ILD and 11 without. The SSc population was composed of 3 limited SSc (12%), 15 lcSSc (60%), 2 dcSSc (8%) and 5 sine scleroderma (20%).

Mean (SD) disease duration, calculated from the onset of the first non-Raynaud symptom was 6.7 ± 7.3 years. Mean (SD) clinical scores were 2.0 (0–6) for the Rodnan score and 13 (interquartile 8–12) for the ACR/Eular score (Table 1). Three of the SSc patients had PAH. At the time of evaluation, 13 patients (52%) were receiving immunosuppressive therapy (1 association of hydroxychloroquine (HCQ) and mycophenolate mofetil (MMF), 7 MMF, 4 HCQ and 1 adalimumab). Ten patients were under oral corticosteroids (OCS, 4.7 mg average dosage prednisone equivalent). There was no significant difference between the two groups (p > 0.05).

**Figure 2:**
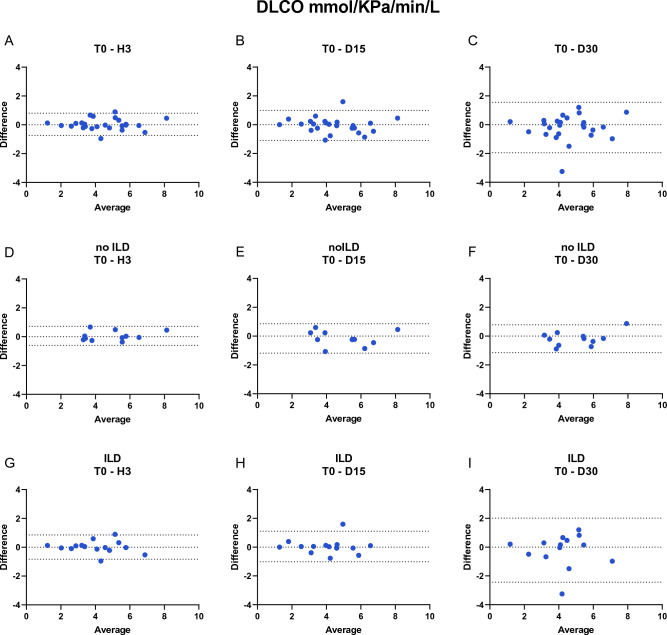
BLAND ALTMAN test DLCO (MM/KPa/min/L).

Interactions between time and group were not significant and are not reported (p > 0.05). The models described test the effect of time and group. Time and group did not significantly influence the different measures of the PFT: FVC [p values time and group effect respectively (0.33; 0.34)], FEV1/FVC ratio (0.093; 0.056) and DLCO (0.99; 0.13) in the ILD and non ILD group (Table S2). We also used a Bland Altman test to assess reproducibility for FVC (L) and DLCO (mMKpa/min/L), Figs. 1 and 2 respectively. The measurements were therefore reproducible over time and in each group.

## Discussion

Our data show that the main longitudinal functional parameters used in the follow-up of SSc patients (FVC, FEV1/FVC ratio, DLCO and KCO) whether suffering from ILD or not, are reproducible over a 30-day study period in a stable-disease population. This study attests to the high reliability of the PFT values for monitoring SSc-associated lung disease.

The reproducibility of PFTs has already been studied in different conditions.

Indeeed, Enright et al. assess the limits for repeatability of FEV1, FVC, and PEF during spirometry test sessions in 18,000 adult outpatients. Within-test variability and daily repeatability has been reported to be ~ 5% in adults^14^. It is usually greater in children up to 10% in young children^20^, although some studies with careful quality control and trained subjects have reported within-test variability values as low as 2% in school-aged children^21^.

In cystic fibrosis population, Inter-session reproducibility data are lower than those previously reported in healthy subjects. Radtke et al. relate the intra-session variability of DLNO, DLCO, DMCO and Vc improves with breath-hold maneuver training in test-naïve patients with CF, indicating a learning effect^12^.

In COPD et asthma population, Xu et al. have demonstrated that repeatability of spirometry parameters was lower than impulse oscillometry resistance parameters in different GOLD (the Global Initiative for Chronic Obstructive Lung Disease) stages, the higher the stage the worse the repeatability^13^.

The results of the present study reflect the characteristics of a sample of SSc patients with stable-disease, which can be considered as a limitation of this investigation. We did not include severe SSc-ILD or patients suffering from severe PAH due to practical issues for the patients. Note that a recent study reports that global survival of PAH-SSc patients was not affected by ILD regardless its severity^22^. Therefore, it is recommended to carry out further research in this line.

The main limitation of our study is the small sample size of the population. Nevertheless, since data on 25 patients did not show significant statistical variability in PFTs, we did not enlarge the population. It should be also noted that these are rather early forms of scleroderma (20% of sine scleroderma), easier to include in the study than stable chronic patients, not wishing to go to the hospital during covid-19 period.

We recall that no therapeutic modification was made during the study period (exclusion criterion). The treatment was thus not a limiting factor.

The best surrogate marker for the onset and progression of SSc-ILD seems to be a composite outcome consisting of a combination of two or more PFT measurements. They could be combined with different blood and lung biomarkers known in SSc^23–26^ to increase sensitivity especially in the early onset of the disease. In this context, specific dedicated composite biomarkers need to be evaluated in order to predict lung disease progression.

Pulmonary function testing remains an important diagnostic tool to monitor patients with SSc-ILD in order to identify early disease progression. Indeed, there is a place for a systematic screening of this population to potentially enable treatment prior to deterioration of lung function in subjects at high risk of disease progression. PFTs should therefore be performed regularly following SSc diagnosis to detect changes related to the occurrence of ILD. Besides, important therapeutic trials as INBUILD and SENSCIS, led to the Marketing Authorization of nintedanib are based in particular on the monitoring of FVC^27,28^. Moreover, recent encouraging data shows in a real-life clinical scenario shows that NTD in combination with immunosuppressants, may stabilize lung function^29^.

## Conclusions

This study confirms that pulmonary function testing, by measuring FVC, FEV1/FVC ratio, DLCO and KCO parameters, is a reliable tool and is not associated with a significant inter-measurement variation during a 30-days longitudinal follow up in a clinically stable SSc cohort with or without ILD. The test–retest reliability is obvious underlying the reproducibility of respiratory parameters and their further use in routine clinical practice, for managing SSc patients.

### Supplementary Information

Supplementary Information.

## Data Availability

The datasets used and/or analysed during the current study available from the corresponding author on reasonable request.

## Abbreviations

ACR: American college of rheumatology
ATS: American thoracic society
AZA: Azathioprine
COPD: Chronic obstructive pulmonary disease
CS: Corticosteroid
D15: T0 + 15 days
D30: T0 + 30 days
DLCO: Diffusing capacity of CO
ERS: European respiratory society
EULAR: European league against rheumatism
FEV1: Forced expiratory volume in one second
FVC: Forced vital capacity
H3: T0 + 3 h
HCQ: Hydroxychloroquine
HRCT: High resolution thoracic computed tomography
KCO: Carbon monoxide transfer coefficient
ILD: Interstitial lung disease
MMF: Mycophenolate mofetil
MTX: Methotrexate
OCS: Oral corticosteroids
PAH: Pulmonary arterial hypertension
PFT: Pulmonary function test
SD: Standard deviation
SSc: Systemic sclerosis
T0: Time 0
lcSSc: Limited cutaneous systemic sclerosis
dcSSc: Diffuse cutaneous systemic sclerosis
TLC: Total lung capacity
VA: Alveolar volume
