# Supplementary material for: Reproducibility of pulmonary function tests in patients with systemic sclerosis
Source: Sci Rep. 2023 Nov 3;13:18960. doi: 10.1038/s41598-023-45881-y (PMC10624913; doi:10.1038/s41598-023-45881-y)
Supplement: Supplementary file 1 — Supplementary Information. [file 41598_2023_45881_MOESM1_ESM.docx]

**Supplementary material**

**Table S1.** Description of the PTFs measurements

| **Variable** | **Time** | **Non ILD patients (N=11)** | | |  | **ILD patients (N=14)** | | |
| --- | --- | --- | --- | --- | --- | --- | --- | --- |
|  |  | **Mean ± SD** | **Median (Q1 ; Q3)** | **Extreme values** |  | **Mean ± SD** | **Median (Q1 ; Q3)** | **Extreme values** |
| FVC (L) |  |  |  |  |  |  |  |  |
|  | T0 | 2.80 ± 0.92 | 2.60 (1.97 ; 3.32) | 1.90 ; 4.43 |  | 2.42 ± 0.73 | 2.34 (1.92 ; 2.86) | 1.48 ; 4.16 |
|  | H3 | 2.88 ± 0.89 | 2.63 (2.12 ; 3.45) | 1.99 ; 4.47 |  | 2.42 ± 0.73 | 2.32 (1.88 ; 2.96) | 1.49 ; 4.09 |
|  | J15 | 2.86 ± 0.81 | 2.80 (2.08 ; 3.16) | 1.96 ; 4.48 |  | 2.37 ± 0.68 | 2.34 (1.96 ; 2.83) | 1.44 ; 3.87 |
|  | J30 | 2.80 ± 0.92 | 2.42 (1.92 ; 3.49) | 1.80 ; 4.44 |  | 2.35 ± 0.73 | 2.29 (1.69 ; 2.69) | 1.51 ; 4.13 |
|  |  |  |  |  |  |  |  |  |
| FVC (%) |  |  |  |  |  |  |  |  |
|  | T0 | 83.9 ± 18.3 | 85.0 (73.0 ; 99.0) | 56.0 ; 117.0 |  | 78.2 ± 20.1 | 72.0 (63.0 ; 88.0) | 56.0 ; 122.0 |
|  | H3 | 85.9 ± 17.6 | 83.0 (74.0 ; 102.0) | 58.0 ; 119.0 |  | 77.6 ± 20.4 | 71.0 (61.0 ; 90.0) | 57.0 ; 119.0 |
|  | J15 | 86.0 ± 16.7 | 81.0 (75.0 ; 102.0) | 63.0 ; 117.0 |  | 78.5 ± 19.8 | 74.5 (67.0 ; 91.0) | 43.0 ; 121.0 |
|  | J30 | 84.2 ± 20.0 | 86.0 (70.0 ; 101.0) | 55.0 ; 121.0 |  | 76.3 ± 18.9 | 68.5 (64.0 ; 81.0) | 56.0 ; 122.0 |
|  |  |  |  |  |  |  |  |  |
| Tiffeneau (%) |  |  |  |  |  |  |  |  |
|  | T0 | 76.2 ± 11.5 | 81.0 (71.0 ; 84.0) | 53.0 ; 86.0 |  | 83.6 ± 5.4 | 84.5 (82.0 ; 87.0) | 74.0 ; 93.0 |
|  | H3 | 76.6 ± 11.6 | 81.0 (74.0 ; 84.0) | 52.0 ; 88.0 |  | 84.1 ± 8.8 | 84.0 (81.0 ; 87.0) | 71.0 ; 109.0 |
|  | J15 | 75.1 ± 12.1 | 79.0 (75.0 ; 83.0) | 50.0 ; 84.0 |  | 83.0 ± 5.7 | 84.0 (78.0 ; 87.0) | 72.0 ; 93.0 |
|  | J30 | 75.5 ± 12.2 | 79.0 (76.0 ; 82.0) | 50.0 ; 86.0 |  | 81.6 ± 6.3 | 82.5 (76.0 ; 86.0) | 69.0 ; 93.0 |
|  |  |  |  |  |  |  |  |  |
| DLCO (mmol/kPa/min) |  |  |  |  |  |  |  |  |
|  | T0 | 4.49 ± 1.61 | 5.37 (3.40 ; 5.79) | 3.19 ; 8.36 |  | 4.06 ± 1.54 | 4.10 (2.92 ; 5.52) | 1.28 ; 6.61 |
|  | H3 | 4.91 ± 1.54 | 4.91 (3.40 ; 5.76) | 3.36 ; 7.90 |  | 4.05 ± 1.59 | 4.37 (2.82 ; 4.93) | 1.16 ; 7.14 |
|  | J15 | 5.13 ± 1.66 | 5.61 (3.60 ; 6.65) | 2.96 ; 7.90 |  | 4.01 ± 1.54 | 4.15 (3.23 ; 4.63) | 1.28 ; 6.51 |
|  | J30 | 5.15 ± 1.42 | 5.42 (3.79 ; 6.24) | 3.13 ; 7.49 |  | 4.27 ± 1.57 | 4.16 (3.59 ; 5.34) | 1.07 ; 7.59 |
|  |  |  |  |  |  |  |  |  |
| DLCO (%) |  |  |  |  |  |  |  |  |
|  | T0 | 68.7 ± 19.1 | 59.0 (53.0 ; 86.0) | 48.0 ; 105.0 |  | 57.9 ± 18.9 | 56.5 (47.0 ; 72.0) | 22.0 ; 87.0 |
|  | H3 | 69.1 ± 17.6 | 62.0 (57.0 ; 83.0) | 44.0 ; 104.0 |  | 57.5 ± 18.1 | 57.5 (49.0 ; 71.0) | 20.0 ; 85.0 |
|  | J15 | 70.7 ± 23.6 | 70.0 (49.0 ; 89.0) | 45.0 ; 121.0 |  | 56.3 ± 21.1 | 52.5 (46.0 ; 70.0) | 22.0 ; 96.0 |
|  | J30 | 68.6 ± 20.6 | 67.0 (48.0 ; 84.0) | 43.0 ; 112.0 |  | 58.6 ± 19.7 | 57.0 (50.0 ; 70.0) | 17.0 ; 102.0 |
|  |  |  |  |  |  |  |  |  |
| KCO (mmol/kPa/min/l) |  |  |  |  |  |  |  |  |
|  | T0 | 1.18 ± 0.28 | 1.12 (0.97 ; 1.40) | 0.77 ; 1.66 |  | 1.17 ± 0.31 | 1.17 (1.08 ; 1.29) | 0.44 ; 1.87 |
|  | H3 | 1.14 ± 0.23 | 1.15 (0.92 ; 1.42) | 0.87 ; 1.49 |  | 1.18 ± 0.28 | 1.20 (1.09 ; 1.24) | 0.42 ; 1.69 |
|  | J15 | 1.19 ± 0.32 | 1.25 (0.93 ; 1.48) | 0.75 ; 1.76 |  | 1.07 ± 0.31 | 1.12 (0.91 ; 1.24) | 0.44 ; 1.63 |
|  | J30 | 1.21 ± 0.24 | 1.19 (0.99 ; 1.43) | 0.88 ; 1.66 |  | 1.13 ± 0.29 | 1.13 (1.08 ; 1.28) | 0.35 ; 1.62 |
|  |  |  |  |  |  |  |  |  |
| KCO (%) |  |  |  |  |  |  |  |  |
|  | T0 | 82.3 ± 19.9 | 80.0 (69.0 ; 97.0) | 54.0 ; 118.0 |  | 82.9 ± 20.2 | 82.5 (76.0 ; 96.0) | 31.0 ; 122.0 |
|  | H3 | 79.7 ± 16.2 | 82.0 (67.0 ; 98.0) | 58.0 ; 105.0 |  | 83.9 ± 19.0 | 84.0 (79.0 ; 89.0) | 30.0 ; 110.0 |
|  | J15 | 83.0 ± 23.6 | 82.0 (66.0 ; 103.0) | 52.0 ; 124.0 |  | 74.4 ± 20.3 | 81.0 (67.0 ; 86.0) | 31.0 ; 114.0 |
|  | J30 | 81.8 ± 20.9 | 85.0 (60.0 ; 99.0) | 50.0 ; 119.0 |  | 78.3 ± 17.3 | 81.0 (76.0 ; 87.0) | 25.0 ; 101.0 |

**Table S2.** Effects of time and group on PTF measurements

| **Variables** | **Time** | **Groups** | | **p-values** | |
| --- | --- | --- | --- | --- | --- |
|  |  | **non ILD (N=11)**  **Mean ± SD** | **ILD (N=14)**  **Mean ± SD** | **Time effect** | **Group effect** |
| FVC (L) |  |  |  | 0.21 | 0.17 |
|  | T0 | 2.80 ± 0.92 | 2.42 ± 0.73 |  |  |
|  | H3 | 2.88 ± 0.89 | 2.42 ± 0.73 |  |  |
|  | J15 | 2.86 ± 0.81 | 2.37 ± 0.68 |  |  |
|  | J30 | 2.80 ± 0.92 | 2.35 ± 0.73 |  |  |
|  |  |  |  |  |  |
| FVC (%) |  |  |  | 0.33 | 0.34 |
|  | T0 | 83.9 ± 18.3 | 78.2 ± 20.1 |  |  |
|  | H3 | 85.9 ± 17.6 | 77.6 ± 20.4 |  |  |
|  | J15 | 86.0 ± 16.7 | 78.5 ± 19.8 |  |  |
|  | J30 | 84.2 ± 20.0 | 76.3 ± 18.9 |  |  |
|  |  |  |  |  |  |
| Tiffeneau (%) |  |  |  | 0.093 | 0.056 |
|  | T0 | 76.2 ± 11.5 | 83.6 ± 5.4 |  |  |
|  | H3 | 76.6 ± 11.6 | 84.1 ± 8.8 |  |  |
|  | J15 | 75.1 ± 12.1 | 83.0 ± 5.7 |  |  |
|  | J30 | 75.5 ± 12.2 | 81.6 ± 6.3 |  |  |
|  |  |  |  |  |  |
| DLCO (mmol/kPa/min) |  |  |  | 0.39 | 0.13 |
|  | T0 | 4.49 ± 1.61 | 4.06 ± 1.54 |  |  |
|  | H3 | 4.91 ± 1.54 | 4.05 ± 1.59 |  |  |
|  | J15 | 5.13 ± 1.66 | 4.01 ± 1.54 |  |  |
|  | J30 | 5.15 ± 1.42 | 4.27 ± 1.57 |  |  |
|  |  |  |  |  |  |
| DLCO (%) |  |  |  | 0.998 | 0.13 |
|  | T0 | 68.7 ± 19.1 | 57.9 ± 18.9 |  |  |
|  | H3 | 69.1 ± 17.6 | 57.5 ± 18.1 |  |  |
|  | J15 | 70.7 ± 23.6 | 56.3 ± 21.1 |  |  |
|  | J30 | 68.6 ± 20.6 | 58.6 ± 19.7 |  |  |
|  |  |  |  |  |  |
| KCO (mmol/kPa/min/l) |  |  |  | 0.41 | 0.70 |
|  | T0 | 1.18 ± 0.28 | 1.17 ± 0.31 |  |  |
|  | H3 | 1.14 ± 0.23 | 1.18 ± 0.28 |  |  |
|  | J15 | 1.19 ± 0.32 | 1.07 ± 0.31 |  |  |
|  | J30 | 1.21 ± 0.24 | 1.13 ± 0.29 |  |  |
|  |  |  |  |  |  |
| KCO (%) |  |  |  | 0.25 | 0.81 |
|  | T0 | 82.3 ± 19.9 | 82.9 ± 20.2 |  |  |
|  | H3 | 79.7 ± 16.2 | 83.9 ± 19.0 |  |  |
|  | J15 | 83.0 ± 23.6 | 74.4 ± 20.3 |  |  |
|  | J30 | 81.8 ± 20.9 | 78.3 ± 17.3 |  |  |

**Table S3.** Respiratory function at T0

|  | No-ILD  N=11 | ILD  N=14 |
| --- | --- | --- |
| FEV1/FVC ratio, %pred. | 75.85 ± 11.7 | 83.08 ± 6.55 |
| FVC post-BD, %pred. | 84.85 ± 18.15 | 77.65 ± 19.8 |
| DLCO %pred. | 69.27 ± 20.22 | 57.57 ± 19.45 |
| KCO %pred. | 81.5 ± 20.15 | 79.87 ± 19.2 |

PFT values at T0, T0+3h, D+15 and D+30 are given in supplementary material (Table S1).

**Figure 3.** Evolution of PFTs measurements for ILD and non-ILD patients. Information is summarized using means ± one standard error


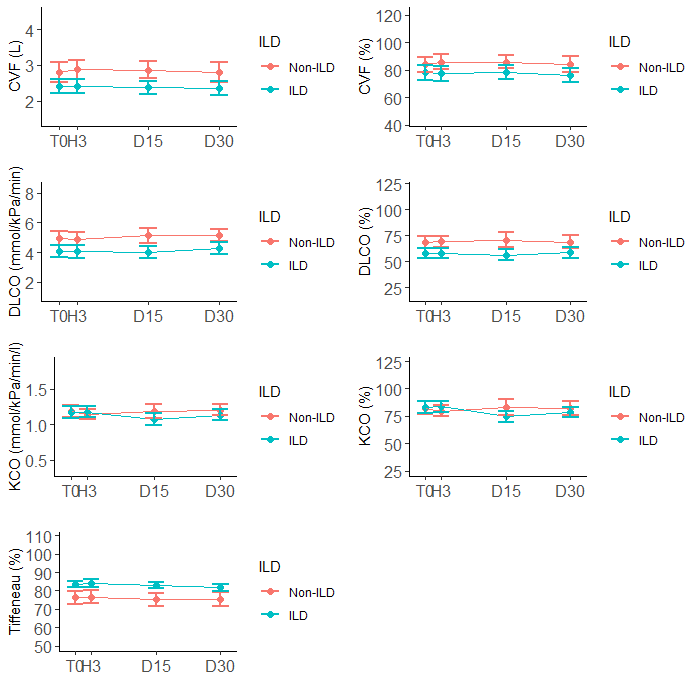


**Table S3.** Bias +/- SD representing average of deviations between T0 and H3 or D15 or D30 +/-SD

|  |  |  | ALL | noILD | ILD |
| --- | --- | --- | --- | --- | --- |
| FVC_L | H3 | Bias ± SD | -0.03±0.1 | -0.0609±0.1155 | -0.005±0.0865 |
|  |  | 95% Limits of Agreement | -0.23 to 0.17 | -0.2872 to 0.1654 | -0.1746 to 0.1646 |
|  | D15 | Bias ± SD | 0±0.17 | -0.0427±0.1518 | 0.0342±0.1766 |
|  |  | 95% Limits of Agreement | -0.33 to 0.33 | -0.3402 to 0.2548 | -0.3118 to 0.3804 |
|  | D30 | Bias ± SD | 0.04±0.15 | 0 ±0.1135 | 0.0721±0.1627 |
|  |  | 95% Limits of Agreement | -0.24 to 0.32 | -0.2224 to 0.2224 | -0.2468 to 0.3911 |
|  |  |  |  |  |  |
| FVC % | H3 | Bias ± SD | -0.52±3.7 | -2±2.86 | 0.64±3.95 |
|  |  | 95% Limits of Agreement | -7.77 to 6.73 | -7.61 to 3.61 | -7.11 to 8.39 |
|  | D15 | Bias ± SD | -1.08±4.98 | -2.09±2.98 | -0.29±6.12 |
|  |  | 95% Limits of Agreement | -10.85 to 8.69 | -7.94 to 3.75 | -12.28 to 11.71 |
|  | D30 | Bias ± SD | 0.96±6 | -0.27±2.49 | 1.93±7.7 |
|  |  | 95% Limits of Agreement | -10.79 to 12.71 | -5.16 to 4.62 | -13.16 to 17.02 |
|  |  |  |  |  |  |
| DLCO_L | H3 | Bias ± SD | 0.0296±0.3945 | 0.0555±0.3376 | 0.0113±0.4295 |
|  |  | 95% Limits of Agreement | -0.7436 to 0.8028 | -0.6062 to 0.7171 | -0.8305 to 0.8532 |
|  | D15 | Bias ± SD | -0.046±0.5319 | -0.1636±0.5192 | 0.0464±0.5422 |
|  |  | 95% Limits of Agreement | -1.088 to 0.9965 | -1.181 to 0.854 | -1.016 to 1.109 |
|  | D30 | Bias ± SD | -0.1992±0.8947 | -0.1836±0.4917 | -0.2114±1.136 |
|  |  | 95% Limits of Agreement | -1.953 to 1.554 | -1.147 to 0.7801 | -2.439 to 2.016 |
|  |  |  |  |  |  |
| DLCO_% | H3 | Bias ± SD | 0.08±6.04 | -0.36±6.23 | 0.43±6.1 |
|  |  | 95% Limits of Agreement | -11.76 to 11.92 | -12.58 to 11.85 | -11.52 to 12.38 |
|  | D15 | Bias ± SD | 0.04±8.14 | -2±7.81 | 1.64±8.32 |
|  |  | 95% Limits of Agreement | -15.92 to 16 | -17.31 to 13.31 | -14.66 to 17.94 |
|  | D30 | Bias ± SD | -0.32±14.05 | 0.09±7.89 | -0.64±17.78 |
|  |  | 95% Limits of Agreement | -27.85 to 27.21 | -15.38 to 15.56 | -35.49 to 34.2 |
|  |  |  |  |  |  |
